# The constrained disorder principle emphasizes the importance of variability boundaries for systems to function effectively

**DOI:** 10.25122/jml-2025-0063

**Published:** 2025-11

**Authors:** Yaron Ilan

**Affiliations:** 1 Faculty of Medicine, Hebrew University, Jerusalem, Israel; 2 Department of Medicine, Hadassah Medical Center, Jerusalem, Israel

**Keywords:** disorder, randomness, variability, complex systems, algorithms, CDP, Constrained Disorder Principle, AI, Artificial Intelligence, HRV, Heart Rate Variability

## Abstract

The Constrained Disorder Principle (CDP) defines systems by their inherent disorder, which is bounded by dynamic borders. This principle determines a system's functionality and efficiency based on its continuously changing boundaries. In this paper, we present the formulation of the principle using the equation **B = F**, where B represents the dynamic borders, and F denotes the system’s function. This equation suggests that the dynamic borders shape a system’s existence, functionality, and efficiency. However, these borders impose a limit beyond which the system cannot further enhance its performance. When disorder surpasses established limits, the system's efficiency begins to decline. Conversely, insufficient disorder may also be harmful in certain situations. The paper examines the causal relationship between disorder and function, illustrating how the equation reflects the system's adaptability, efficiency, learning capabilities, memory, energy consumption, aging, and eventual termination. We also discuss how this formula can be applied to correct malfunctions and enhance system functions. Furthermore, we introduce a second-generation artificial intelligence system based on the CDP formula that incorporates noise. In summary, the B = F equation provides a valuable framework for understanding complex systems and lays the groundwork for models designed to enhance system performance.

## Introduction

In ancient history, randomness and chance were associated with fate [1,2]. Determinism is a philosophical view in which previously existing causes determine all events. Most biologists support deterministic interpretations of phenotypic variation patterns [3]. Heisenberg's indeterministic uncertainty principle led to a rebuke of the laws of physical causality and to the presence of indeterminism in physics and biology [4]. In the 1980s, it was confirmed that Heisenberg, Bohr, and others were correct regarding the uncertainty principle. Complex systems do not operate in a deterministic manner, and at the atomic and subatomic levels, the only thing definite is a certain degree of randomness [5].

Natural systems, randomness, and rules coexist. Previous works highlighted the inherent randomness of complex systems in the universe. This randomness was demonstrated at the genome level and in whole-organ functions in biological systems [6-9].The constrained disorder principle (CDP) defines systems as characterized by their inherent disorder limited by dynamic boundaries [10].

The paper outlines the principle and discusses its implications for complex systems. It also presents the development of regimens based on the CDP formula to enhance the functions of systems powered by second-generation artificial intelligence (AI).

## The constrained disorder principle

### The constrained disorder principle outlines the system's function

The constrained disorder principle states that randomness and dynamics are inherent to all systems and are constrained by dynamic borders, which are also variable [10]. The CDP defines a system's degree of functionality and efficiency in terms of its inherent disorder. Per the CDP, the system's function is determined by its degree of variability. The higher the variability, the higher the efficacy, as long as it remains within the dynamic limits [10].

The principle formulation is given by the **B = F** formula, where B stands for the *borders* and F for the *function*.

The formula implies that the function of any system is defined by its borders. Borders are the constraints on the disorder of systems. The wider the borders are, the more the disorder within a system implies improved function. Increased disorder implies better efficiency within dynamically changing borders. However, the border limit is beyond which systems cannot improve further. Thus, the system loses efficiency if the disorder is outside its borders.

The **B = F** is also **B = E**, where E stands for existence, implying that the borders define the system’s existence. E also stands for *efficiency* and *effectiveness*, indicating that the borders determine the amount of energy produced and used by a system.

**Figure 1:**
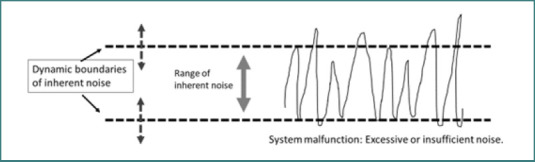
Dynamic boundaries define the noise range within a system, enabling it to adapt to internal and external perturbations and ensure its functionality. The noise range within a system is dynamic and continuously adjusts to accommodate both internal and external disturbances. Insufficient noise or noise outside acceptable boundaries is associated with potential system malfunctions.

Figure 1 schematically illustrates the role of dynamic boundaries in determining the noise range within a system, enabling the system to adapt to internal and external perturbations and ensuring its functionality.

### The B = F formula accounts for the system's adaptability, efficiency, learning, memory, time use, energy use, and aging

The B = F formula accounts for the noise that characterizes systems. It provides a simple solution to a highly complex problem, implying that a system's borders define its function under all conditions. If a system fails to adapt appropriately to changes in its internal and external environments and its narrow boundaries are not dynamic, its function declines.

The dynamic nature of borders necessitates continuous, proper adaptation to the environment. According to the CDP, the borders are dynamic and continually change to reflect the system's flexibility and adaptability to ever-changing internal and external triggers. The range and dynamicity of its borders reflect the system's dynamicity and adaptability to its active internal and external environments. The B in the formula represents the degree of variability in the borders. B implies dynamically changing borders, providing upper and lower limits for the system's disorder. If the degree is too low or too high, or if it is constant, the system's function decreases.

The B = F formula determines the system's function of simple and complex systems. The system's efficiency is always proportional to B. The formula implies that quantifying a degree of variability and randomness is mandatory for understanding the system's function and for correcting malfunctions. When evaluating a system's performance, the formula shifts the focus from noise analysis to the examination of augmented responses and outcomes under continuously changing conditions, reflecting the system's adaptability and efficiency in responding to internal and external perturbations.

According to the CDP, all systems are composed of multiple subsystems and processes, and the same laws apply to all levels, including those involving quantum effects. The B = F formula accounts for the inherent randomness of quantum systems. Interactions between systems affect the system's function, as reflected in the formula [11,12].

B reflects a system's degree of variability and its memory. A system's accumulated history, learning capabilities, and ability to respond to internal and external perturbations are embedded in B and are accounted for by the borders' dynamicity. The dynamicity of the borders reflects their memory, accumulated experience, and history. Systems improve with time through their learning experience.

The CDP formula differentiates between living and non-living organisms based on their variability and borders [10]. Living organisms are characterized by a high degree of disorder that continually changes within their dynamic boundaries. Non-living organisms exhibit lower variability and narrower boundaries, and a certain degree of variability in these systems improves their performance [13-16].

The B = F CDP formula accounts for the time dimension. It implies the continuously changing degrees of disorder in the system and the shifting borders over time.

A system's outcome is determined by its function, which reflects its efficiency and the energy it consumes. The B = F formulation illustrates the amount of energy a system utilizes. Working under a certain degree of disorder improves the system's function and efficiency for a similar amount of energy. However, systems that are too disordered or too ordered, and function outside their borders, require higher energy levels to achieve similar functionality. These systems are energy-inefficient [17]. Systems use continuously changing dynamic borders to achieve the lowest energy requirement under environmental changes.

According to the CDP, aging refers to the process of reducing variability in boundaries, resulting in narrower borders, less disorder, and increased variability outside these boundaries. In all instances, this leads to a decline in function. In the context of the CDP system, termination and death signify either a complete loss of order, a significant increase in disorder that goes well beyond established boundaries, or a loss of the dynamic nature of boundaries and their ability to respond to disruptions.

### The complexity of quantifying noise in complex biological systems

Achieving complete accuracy in quantifying the disorder and defining its boundaries is a complex task. The B = F infers that, as borders continuously change, quantifying them at a particular moment does not reflect the overall system behavior.

According to the CDP, in complex systems where multiple variables change randomly and continuously, as in biological systems, the quantification of B may not be sufficiently accurate to provide information about the system, rendering B unquantifiable in such systems [18].

Scientists commonly use statistical methods to overcome variability in experiments and real-world data analysis. However, when variability inherent to complex systems is not adequately addressed, it can lead to incorrect conclusions and misleading statements about the findings [19]. Using means to overcome the inherent variability of systems may result in inaccurate models [19-21]. Methods based on probabilities and distributions can determine the trajectories of systems but cannot accurately quantify the highly dynamic disorder [22,23].

Noise is inherent to both information transmission and development. If stochasticity is vital in biology, it cannot simply be reduced to an average. Noise carries information, and if we ignore it by using a mean, we miss crucial details. A population with diverse phenotypes suggests that the average does not capture the entire picture [13-15,24].

Methods that enable a more precise quantification of disorder enhance the predictability of variables B and F. While achieving closer estimates of B and F with relatively high accuracy is beneficial, it may not always accurately capture the time dimension or the system's dynamic nature. Probabilistic statistical methods can aid in analyzing noisy systems. Approximations and the use of distributions and statistical methods to determine noise in systems are beneficial for understanding the trajectories of these systems. However, they may be insufficient for achieving the required accuracy when dealing with complex biological systems characterized by continuous, unpredictable internal and external noisy perturbations [25,26]. Denoising methods aim to remove "noise" that arises from measurement errors and confounding variables [27,28]. Differentiating between noise, as per the CDP, is mandatory for the proper functioning of systems, and the “undesirable technical noise” is challenging, leading to biases in the output. Methods for smoothing noise are less relevant to systems that require dynamic disorder for their function [29-32].

Complex systems, such as biological systems, are characterized by multiple variables that contribute to inherent, unknown noise; therefore, they cannot be accounted for in modeling [31]. When the outcome involves some variability and systems operate more effectively under dynamic conditions influenced by multiple unknowns, it may be impossible to account for all parameters with complete accuracy. In contrast to physics, where models consist of a limited, well-defined set of measurable variables, the factors contributing to noise in biological systems are countless. In biological systems, a certain level of noise must always be acknowledged, and simply increasing repetitions or gathering more data does not always resolve this issue.

### B = F implies causality between disorder and function

The CDP, reflected by the B = F, implies that a number for B, which is not too high or too low, provides the "ideal range” for a function that requires the least energy. The B = F mandates causality between a constrained disorder and efficiency. The system's dynamicity enables it to function within continuously adapting borders, implying alterations in the degree of disorder to maintain maximal efficiency and reduce energy requirements under multiple internal and external perturbations [11].

Heisenberg’s uncertainty principle, stating that "one can never be sure of anything, and there are some things you can never be sure of,” was initially relevant to microscopic particles and had implications for understanding the universe [33,34]. Heisenberg's principle is derived from a fundamental understanding of quantum mechanics, which applies to all forms of matter and energy. Formulating this principle involves uncertainty in the particle's position and speed, requiring Planck's constant to lie within a range. It did not explicitly specify how one can know something about a system, arguing that pairs of properties, termed canonical conjugate variables, are linked but cannot be measured accurately simultaneously [35]. An example is the pair of time and energy: the more accurately the time frame in which something happens is calculated, the less is known about the energy involved [36,37].

According to the CDP, a system's disorder indicates its functionality. It is not always possible to determine a system's boundaries with complete accuracy, which may leave some uncertainty about them.

A system's function, when measured continuously over time, reflects the degree of variability within the system. While it may not always be possible to quantify the boundaries and degrees of variability with complete accuracy, the trajectory of the system’s outcome is measurable; this does not necessarily imply causality. In cases where causality is clear and exact sequences of events follow one another, one sees the averaged results of multiple submicroscopic encounters, each operating by chance. Similarly, the immense variability characterizing a system reflects the sum of the degrees of variability and disorder inherent to all its parts and processes. While the result of a system's function appears ordered, it is underscored by the constrained disorder of the multiple subsystems that comprise a system [38].

Nature does not eliminate randomness; instead, it creates reliable systems and processes that operate effectively. Stochastic gene expression refers to the random activation of genes. The activity of genes within individual cells is often much more chaotic than it appears from the outside [39,40]. For example, flowers unfold with surprising precision, even though their gene activation is not a linear process. Genes that respond to auxin, a growth hormone, activate unpredictably across different cells, despite being exposed to the same hormone levels. While individual cells may show inconsistencies, groups of cells can average out these variations, resulting in a stable collective signal for plant development [41].

## Using the CDP to overcome system malfunctions

### Use of the B = F formula to improve and correct systems

A system malfunction is viewed as a loss of order and having too high a level of disorder outside the boundaries. The CDP implies that adding noise to systems or attempting to limit disorder when it exceeds the boundaries can improve system function and correct malfunctions. It requires differentiating the inherent noise of systems from the unwanted noise resulting from measurement issues and confounding variables.

The CDP enables the design of an algorithm to determine the most effective way to utilize a system's disorder, variability, and randomness. It is based on continuously quantifying signatures of variability in a system over time. Noise modeling is used to characterize noise structures and analyze their impact [42]. Alterations in a system's noise are used in various models to enhance performance [43]. The yields of these models provide estimates of variability based on several measurable parameters relevant to the outcome. The B = F formula accounts for a system's inherent noise. Quantifying variability signatures provides a means to improve systems' functionality, enabling the addition or reduction of the degree of noise in systems. This method is limited by its inability to accurately measure variability that changes, as well as the challenge of differentiating between required and unwanted noise. Nevertheless, it enables the setting up of platforms based on the best possible measurements [38,43,44]. Bioengineering of complex systems requires introducing disorder to enhance the system’s function [37]. It implies widening the borders to a level that allows more disorder if kept within the boundaries. There are several examples where adding noise or denoising can improve systems' function [45,46]. Nevertheless, noise can be quantified, analyzed, and used to improve function. Adding noise to systems helps regulate the disorder required for proper function [43].

Randomness is certified if it describes events that an external adversary cannot predetermine [41]. Weakly certified randomness can be amplified towards ideal randomness using quantum-mechanical systems. An example is an error-tolerant protocol that uses a finite number of devices to amplify arbitrary weak randomness towards perfect random bits, which are secure against a no-signaling adversary [47,48].

Noise reduction, denoising, removes noise from a signal [49]. Several noise reduction techniques exist, some of which can distort the signal to some degree [43,44]. Adding noise during the process and then denoising is a method that can control the noise that characterizes the end product [49].

Data augmentation is a method that increases the amount of data by adding modified copies of existing data or newly created synthetic data derived from existing data [50]. Data augmentation increases the amount of training data and achieves robustness against noise. A data augmentation method implemented using the adaptive inverse peak signal-to-noise ratio can improve image quality [51]. Adding noise can be used for data augmentation, thereby expanding the training dataset sizes [52,53]. When a training sample is presented to the model, noise is added to the input variables, making them different each time [52].

Biological systems are characterized by inherent noise. Correcting malfunctions and improving functionality requires implementing noise reduction by quantifying noisy variables and denoising unwanted noise. While these are complex systems comprising numerous unknown or unmeasurable variables, using the CDP-based concept of adding noise or denoising based on the best possible assessment of the disorder status provides a means for augmenting function.

Biological noise is a double-edged sword, both beneficial and deleterious [53]. According to the CDP, an optimal level of noise is necessary for improving functionality. It is crucial to differentiate between technical variability and the inherent biological noise that characterizes systems for proper modelling of systems [13-15,54,55].

### Noise can be used to correct the system's malfunction and improve effectiveness

Second-generation AI systems evolved from quantifying signatures of disorder and implementing them into algorithms to improve the systems' functionality. The example below describes a second-generation system to design a therapeutic regimen for patients with chronic diseases [10,56-61].

Patients with chronic diseases, including hypertension, heart disease, diabetes, cancer, neurological diseases, and inflammatory disorders, often develop tolerance and resistance to chronic therapies [62]. Randomness is fundamental to biological systems and is integral to their proper function, from DNA and cellular organelles to organs. For most biological processes, stochastic effects are required in addition to deterministic ones. In most biological systems, regulatory mechanisms control the disorder but do not eliminate it [63,64]. Second-generation AI systems generate therapeutic regimens based on the noise signatures from the different organs to overcome drug resistance.

The example assumes an "ideal" dosing regimen, *C*, at the time. *t_i_* as a function of the patient's clinical outcome in earlier recorded measurements *W_j_* (for *j*<*i*) and earlier dosing *S_j_* (for *j*<*i*), and at time-independent parameters such as gender, *g*, age (at the beginning of the treatment), *a*, ethnicity, *e*, and other parameters. Hence *C_i_*=*C_i_* (*W_j_,S_j_*;*g,a,e*…). The ideal dosing regimen depends on a time series of varying lengths, so the algorithm is formulated using a Recurrent Neural Network (RNN). Because long- and short-term dependencies may arise, the algorithm combines Long- and Short-Term Memory (LSTM) cells in the network. A deep network with a high level of abstraction generates the rules that define a correct treatment regimen.

The presented method is "supervised" learning. The network is trained on data from scenarios to predict the outcome of the proposed treatment. When the result is unclear, the algorithm is trained on single or multiple patient data. As a random treatment regimen within approved dosing ranges and administration times approximates the optimal regimen during the initial treatment period, the algorithm provides a random regimen within predefined boundaries, providing the neural network with its labels while offering patients an improved therapeutic schedule. Once the network is sufficiently trained, the efficiency of a proposed treatment is predicted. *S_i_* given *C_i_* (*W_j_,S_j_*;*g,a,e*…). It enables the calculation of an ideal regimen in a personalized manner [50-52].

The closed-loop system is based on input data comprising physiological data and signatures of variability, which are quantified periodically and incorporated into the algorithm as part of the input. The target clinical outcome is continuously assessed to determine whether a clinical improvement is achieved and maintained over time. Based on that analysis, a new therapeutic regimen is constantly generated to maintain a sustainable response [56].

The dynamic dosing regimen is continually updated in a personalized way, adapting itself to the subject based on the desired outcome. The algorithm determines, in a customized manner, the best signatures of variability that contribute most to the desired clinical result. The selected signatures are incorporated into the upgraded algorithm levels, which continuously learn from measured parameters to improve clinical outcomes [65-93].

A single-subject therapeutic regimen is being developed based on extensive data analysis, and the patient's data comprise a regulated disorder characterized by selected variability signatures. A dynamic, insightful database is generated from data from multiple patients, accounting for outliers from the plurality of users, for whom the learnings of general users may not be applicable, while developing new treatment models for these outliers. The regimen generated by big data is further analyzed for various disease and host-related variables, concomitant diseases, geographic location, concomitant medications, and other parameters, which differ dynamically for each patient. It contributes to the deep learning algorithm that generates a personalized, patient-tailored treatment regimen. The algorithm continuously learns and adapts based on the data accumulated from big data and that received from each patient [50,51,56].

This example illustrates the complexity of incorporating noise into biological systems. It acknowledges that measured variables in these systems may not be relevant to the outcome and that many variables are unknown, unmeasurable, and continuously changing, which limits the model's ability to achieve high accuracy.

### Three steps for increasing the performance of second-generation AI systems based on the B = F formula

CDP-based second-generation AI systems are developed on three levels to generate treatment regimens for patients with chronic diseases to overcome drug resistance [66-94]. At the first level, the system incorporates variabilities within a predetermined range into the treatment plan, which is non-personalized and independent of the outcome. The physician predefines dosing ranges and administration schedules for patients with chronic diseases who have lost response to their medications. Patients receive an individualized dosing plan using an open-loop system that changes daily in dose and administration times, keeping them within the predetermined treatment dosing plan [56,57].

At the second level, the algorithm implements a closed-loop system. The algorithm's endpoint is a meaningful clinical outcome. The system learns from each subject, large populations, and digital twin systems to identify and generate the ideal treatment regimen for a subject [56,57]. The algorithm continually searches around the optimal dosages and drug delivery times, never keeping them identical, and imposes different constraints on the randomization based on the clinical outcome [56,57]. In cases where some measured variables can be continuously monitored, the algorithm's output is improved by providing a method for more precisely defining B.

At the third level, the algorithm is augmented by quantifying variability signatures related to the disease, the environment, and the host. These are dynamically measured parameters directly or indirectly associated with the disease or the host. Implementing these signatures increases the accuracy of the algorithm output [56,57]. Identifying quantifiable random variables and generating therapeutic regimens based on them stems from the notion that these signatures are inherent to biological systems and essential to their function. It implies getting the algorithm closer to biological randomness. Using approximations is compulsory due to the numerous unquantifiable variables that characterize biology; nevertheless, it provides a means to augment function [58].

An example is heart rate variability (HRV), which measures the variation in the time between heartbeats, controlled by the autonomic nervous system (ANS). HRV is a non-invasive tool for assessing cardiac autonomic regulation and predicting clinical outcomes in patients with cardiovascular disease [94]. HRV is an independent predictor of cardiac or all-cause mortality [95,96]. Patients with congestive heart failure and reduced cardiac function exhibit changes in HRV that are proportional to the severity of their condition. The algorithm utilizes these quantified HRV changes to tailor treatment regimens in a personalized manner.

In patients with inflammatory disorders, fluctuations in cytokine measurements over days are quantified and incorporated into the treatment algorithm to enhance the effectiveness of anti-inflammatory medications. Since the variability patterns and their dynamics are unique to each individual, the algorithm adjusts its recommendations based on individual data while learning from large datasets.

Clinical benefits from using the algorithm have been reported in patients with chronic heart failure, drug-resistant cancer, multiple sclerosis, chronic pain, genetic disorders, and age-related conditions [61,90,97,98].

The example demonstrates how a second-generation AI can augment the functions of complex systems using noise [50,51,56]. Improving the system's function and correcting malfunctions is feasible using the B = F formula by either increasing noise when it is reduced or reducing it where noise is outside the acceptable range. In patients with heart failure, chronic pain, and multiple sclerosis, adding noise to therapeutic regimens improved response to chronic therapies [13-15, 54, 55, 59, 61, 62, 65-92, 97-99].

The innovative CDP-based concepts of harnessing noise to enhance functionality are applicable across various fields, including engineering, economics, sport, and social systems [9,44,55,81]. New research is unfolding, promising to uncover additional possibilities for applying CDP-based systems in these dynamic areas.

### The B = F formula in complex systems: translating variabilities into algorithm outputs: limitations and future directions

Translating quantified variability signatures into treatment algorithms is not intuitive and raises questions for study. It is unclear whether reduced variability necessarily requires a higher-variability regimen or vice versa. It is due to an inability to define the borders accurately.

The CDP presented by the B = F formula raises the question of whether it is advantageous to restrict an algorithm further by quantifying the signatures of disorder. For treatment regimens, when the degree of variability in selected biomarkers increases, it is unclear whether the algorithm's output should increase or decrease dosing variability or the time ranges for drug administration.

Quantifying variability signatures, even over a specific period, may be insufficient, even if measured repeatedly, as it overlooks the time dimension in the functions of complex systems. Associations between quantified variability and a clinical outcome do not necessarily imply that higher or lower degrees of variability should be treated differently. Approximations based on arbitrary measures can enhance understanding of complex systems but may yield biased results. When it is not possible to obtain clinically relevant approximations, an outcome-based randomization regimen can help reduce bias. A process of trial and error of approximations of variability-based algorithms, which adapt themselves based on outcomes, can identify the "close to ideal ranges of variability" [61].

As a constrained disorder is mandatory for proper function, narrowing the borders may not augment effectiveness unless it is clear that the disorder is outside the borders. Using variability signatures relevant to the host or disease, and outcome-based closed-loop systems, is expected to improve the algorithms' approximations and outputs. Further studies are ongoing to dissect these questions in several chronic diseases.

Quantifying variability signatures requires distinguishing between the inherent noise of systems and the unwanted noise arising from the measurement challenge, thereby reducing algorithm accuracy. The differentiation is easier in physics, where variables are accountable. Still, it remains a challenge in biology, where numerous unknown variables exist, and countless known ones cannot be accurately measured [60].

The associations between diverse variabilities, such as genome, cellular organelle, or organ variabilities, and algorithm outcomes are not uniform for all parameters and may require different modeling approaches. As variability is inherent to systems and several variables can be quantified with good approximations, therapeutic regimens that implement random drug administration can be generated based on these signatures. Nevertheless, it does not imply a direct correlation between variability signatures and outcomes.

An unresolved issue under investigation is the degree of personalization of the variability. In a fully randomized system, the degree of disorder is unpredictable and continuously changes. What proportion of the dynamicity in the degree of variability is personalized under different conditions is to be determined. If subjects exhibit varying degrees of variability in a measurable parameter, and these differences are dynamic, their relevance to the algorithm may be minimal.

In complex systems, continuous measurement of the degree of variability is not feasible, making it challenging to accurately adapt the output to varying degrees of variability. Nonetheless, the input may not necessarily be directly linked to the outcome. It implies there may be no need to quantify the variabilities, as these may be irrelevant inputs for improving the outcome. Keeping an irregular output independent of the algorithm's input may improve outcomes when inputs cannot be translated into a continuous dynamic (e.g., administering a drug twice a day).

Explaining complex systems with multiple variables based on the B = F formula involves examining the sum of the functions and variabilities of all parts of subsystems. Malfunctions of the immune system can manifest as inflammation or exhaustion when variability is too low or too high. A cancerous process reflects increased disorder or a lack of regulation, but may also be associated with reduced disorder [95]. Oncogenic mutations may become cancerous to increase their entropy [100]. According to the CDP, this implies being outside the borders but does not rule out the need for a certain degree of randomness. The B = F formula reflects the relationship between the system's inherent variability and its function. However, the mechanisms by which the disorder improves function are being studied. A certain degree of disorder characterizes systems, but it does not explain causality.

## Conclusion

The B = F formula describes the CDP and offers a framework for better understanding systems. This formula encompasses all aspects of a system's features and functions, considering the processes that define both complex and simpler systems. It facilitates the design of innovative methods to correct system malfunctions and enhance their performance. By applying this design formula and conducting experiments, challenges in studying complex systems can be addressed effectively. Ongoing research focuses on utilizing this formula to enhance system performance and develop more accurate digital twins.
